# A Computational Biology Study on the Structure and Dynamics Determinants of Thermal Stability of the Chitosanase from *Aspergillus fumigatus*

**DOI:** 10.3390/ijms24076671

**Published:** 2023-04-03

**Authors:** Qian Wang, Song Liu, Kecheng Li, Ronge Xing, Xiaolin Chen, Pengcheng Li

**Affiliations:** 1 CAS and Shandong Province Key Laboratory of Experimental Marine Biology, Center for Ocean Mega-Science, Institute of Oceanology, Chinese Academy of Sciences, Qingdao 266071, China; wangqian201@mails.ucas.ac.cn (Q.W.);; 2 Laboratory for Marine Drugs and Bioproducts, Pilot National Laboratory for Marine Science and Technology (Qingdao), No. 1 Wenhai Road, Qingdao 266237, China; 3 University of Chinese Academy of Sciences, Beijing 100049, China

**Keywords:** chitosanase, thermal stability, structure prediction, dynamics simulation, binding free energy

## Abstract

Environmentally friendly and efficient biodegradation with chitosanase for degrading chitosan to oligosaccharide has been gaining more importance. Here, we studied a chitosanase from *Aspergillus fumigatus* with potential for production, but does not have the ideal thermal stability. The structure predicted by the Alphafold2 model, especially the binding site and two catalytic residues, has been found to have a high similarity with the experimental structure of the chitosanase V-CSN from the same family. The effects of temperature on structure and function were studied by dynamic simulation and the results showed that the binding site had high flexibility. After heating up from 300 K to 350 K, the RMSD and RMSF of the binding site increased significantly, in particular, the downward shift of loop6 closed the binding site, resulting in the spatial hindrance of binding. The time proportions of important hydrogen bonds at the binding site decreased sharply, indicating that serious disruption of hydrogen bonds should be the main interaction factor for conformational changes. The residues contributing energetically to binding were also revealed to be in the highly flexible region, which inevitably leads to the decrease in the activity stability at high temperature. These findings provide directions for the modification of thermal stability and perspectives on the research of proteins without experimental structures.

## 1. Introduction

Chitosan is a kind of alkaline polysaccharide in nature that is obtained by the deacetylation of chitin and is comprised of β-(1→4)-linked d-glucosamine and *N*-acetyl-d-glucosamine units [1]. Chitosan usually has poor solubility due to its high molecular weight, but the chitosan oligosaccharide with a lower molecular weight obtained after degradation can have better solubility. Aside from the its non-toxic and good biocompatibility, when the molecular weight is degraded to low enough, the chitosan oligosaccharide shows many good properties such as antibacterial and anticancer [2,3], which can be widely used in the food, pharmaceutical, agriculture, and environmental protection fields [1,4,5,6,7]. Therefore, whether chitosan can be effectively degraded is an important aspect for its application. Compared with traditional chemical and physical degradation methods, enzymatic degradation is gaining attention because it does not cause pollution, has a narrow molecular weight distribution, and its mild reaction condition is easier to satisfy [3,8,9].

Chitosanase, which catalyzes degradation reactions, is usually produced by bacteria and fungi [10,11,12]. The endo-chitosanases identified so far are commonly found in families GH5, 7, 8, 46, 75, and 80, while exo-chitosanases are found in the families GH2 and 35 [13,14]. Among the endo-chitosanases, the GH46, GH75, and GH80 families are specific to chitosanase, while the GH5, GH7, and GH8 families also have other enzyme activities such as cellobiohydrolase, glucanase, etc., which belong to non-specific enzymes. The specificity of chitosan degradation by chitosanase may be due to the fact that only the amino group in chitosan can interact with certain catalytic residues, resulting in a strong affinity [15]. The optimum reaction temperature of most microbial chitosanase is between 50 and 70 °C, and the optimum pH range is generally between 4.0 and 8.0. Since many enzymes are derived from normal-temperature bacteria, they have fully adapted to the catalytic conditions determined by the environment during the process of evolution. Therefore, their stabilities are generally poor under the high temperature conditions of industrial production, which seriously restricts their application. The focus of this paper was on chitosanase from the fungus *Aspergillus fumigatus*, which is widely distributed in terrestrial and marine environments. In a previous study [16], the chitosanase from *Aspergillus fumigatus* Y2K was purified and determined to have a molecular weight of 25 KDa and could degrade chitosan to produce oligosaccharides with degrees of polymerization (DP) from DP2 to DP5. Its optimum reaction pH and optimum temperature values were 6.5 and 65–70 °C, respectively, but the enzyme activity was relatively stable below 55 °C, which means that it cannot maintain enzyme activity for long at the optimum temperature. In another study [12], the chitosanase was heterologously expressed in *E. coli.* Its sequence, which was numbered Q875I9 in the CAZy database [17], was determined to have a total of 238 residues and has been identified as belonging to GH75. Residues Asp160 and Glu169 (Asp143 and Glu152 after excision of the signal peptide) were shown to be its catalytic residues by targeted mutagenesis, since substitution of either of them resulted in a complete loss of enzyme activity and was not caused by changes in the protein structure. Similar results were seen in the studies of two other GH75 family members. Shimosaka found that ASP175 and GLU188 are necessary catalytic residues of the chitosanase from *Fusarium solani* by mutagenesis [18]. Wu identified the catalytic residues as ASP148 and GLU157 in the chitosanase from soil virus AMG by the same method, and found that these two residues were universally conserved in the GH75 family [19].

The structure of a protein determines its function, and loss of function is often caused by changes in structure. We hypothesized that high temperature may cause some significant changes in the structure of chitosanase, hindering the binding with ligand and leading to an obvious decrease in the activity stability. Therefore, we began a study on the effect of temperature on thermal stability on the basis of structure. The structures of many families of chitosanase (such as GH8 and GH46) have been determined and well-studied [11,15,20,21,22], but only one member from GH75 has recently been determined [19]. Therefore, we used Alphafold2 [23] to predict the structure, and verified the predicted structure by evaluation tools, molecular docking, and experimental findings.

The simulation method based on molecular dynamics (MD) simulates the motion of the substances from a microscopic point of view, which is very effective in studying dynamic properties such as protein structure change, enzyme activity, ligand recognition, and binding [24,25,26,27]. According to the literature reports [16], Q875I9 exhibited significant differences in enzymatic stability between above and below 55 °C, and its optimum catalytic temperature was below 70 °C, indicating that the negative effect brought by heating above 70 °C began to exceed the positive effect. The conformational changes may be very worthy of attention. To fully sample the possible conformations under different conditions, we chose two temperatures, 300 K and 350 K, for the dynamic simulations to capture the significant differences between conformations. RMSD, RMSF, and the radius of gyration were calculated to analyze the overall and local fluctuations of the enzyme at different temperatures, and dynamic cross correlation was used to research the correlation of original motion, and PCA and cluster analysis helped us to find the dominant conformations. Molecular interactions were analyzed to determine the factors responsible for conformational changes and the thermodynamic correlation between temperature change, and the thermal stability was explored by calculating the binding free energy.

## 2. Results and Discussion

### 2.1. Structure Prediction and Evaluation

**Figure A1:**
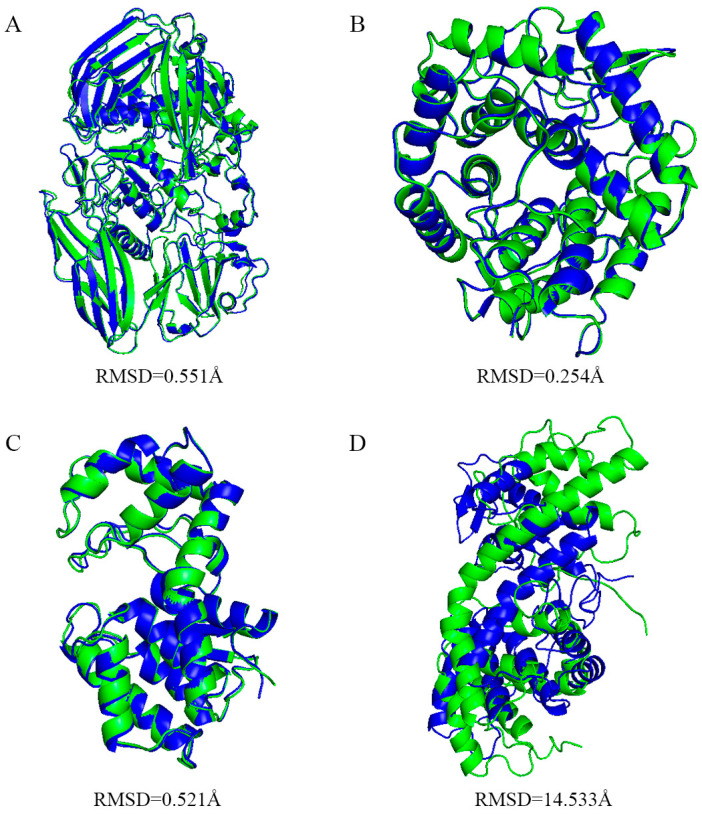
Predicted structures of four chitosanases. (**A–D**) show the results of aligning the predicted structure of the chitosanase from GH2(ID: 2x05), GH8(ID: 5xd0), GH46(ID: 4olt) and GH80(ID: 5b4s) to the experimental structure respectively.

Alphafold2 has been used to predict V-Csn from the Gh75 family and has obtained excellent prediction accuracy (only 0.6 Å error in align with experimental structure) [19]. Despite the previous excellent performance, we still validated the prediction ability of Alphafold2 in the classification of chitosanase beforehand. We predicted four chitosanase with experimental structures from the families GH2(ID: 2x05), GH8(ID: 5xd0), GH46(ID: 4olt), and GH80(ID: 5b4s), and then aligned the predicted structures with the experimental structures using Pymol [28] to examine the prediction accuracy (Figure A1). Three enzymes from the families GH2, 8, and 46 all obtained high accuracy prediction results (RMSD below 0.6 Å), the errors were mainly located in loop regions with high flexibility, and the predicted lDDT [23] were above 95 accordingly. The prediction error for the enzyme from GH80 was larger (RMSD above 14 Å), while the predicted lDDTs were also generally below 60, reminding us of the low confidence in the prediction. This indicates that although the model cannot provide the correct structures for all amino acid sequences, the confidence scores are still reliable.

**Figure 1:**
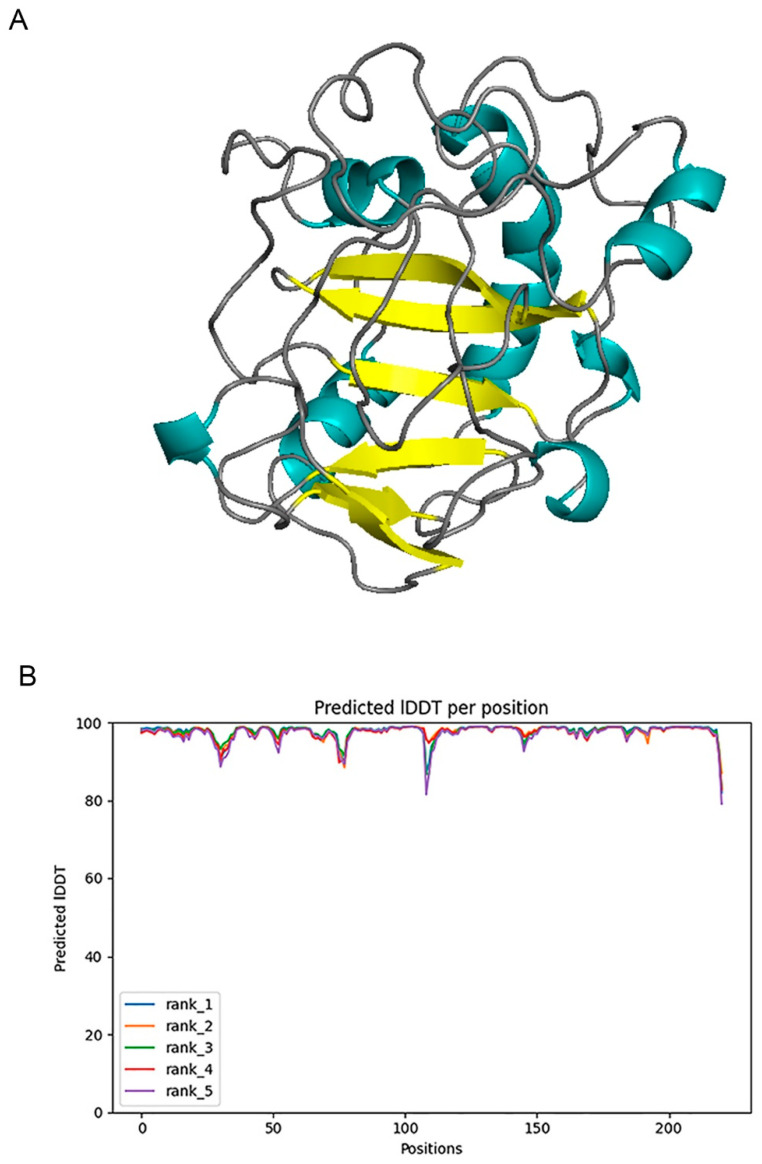
(**A**) 3D structure of chitosanase Q875I9 predicted by Alphafold2 (cyan represents the helix, yellow represents the sheet, gray represents the loop). (**B**) The predicted lDDT of five predicted structures given by Alphafold2.

**Figure 2:**
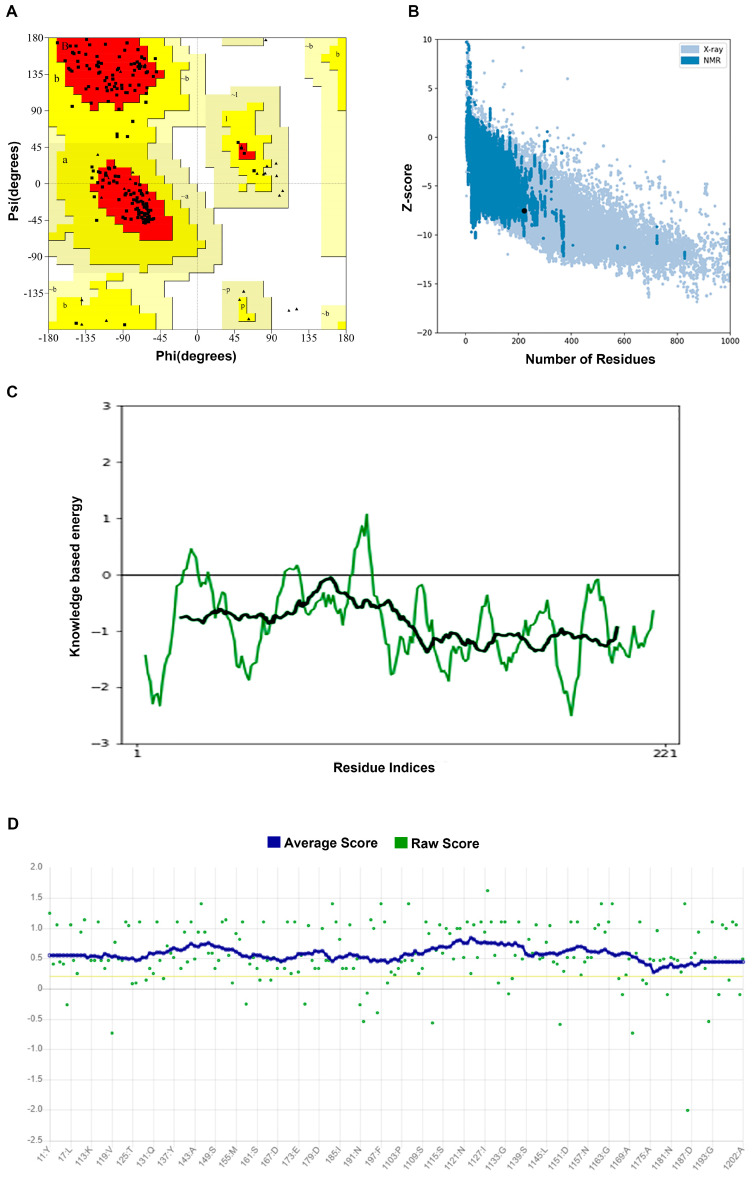
(**A**) Ramachandran plot of the predicted structure calculated by PROCHECK. Red represents the most favored regions and yellow represents the allowed regions; the model can be regarded as high quality when about 90% of the rotations are in red. A, B, L: most favorite regions. a, b, l, p: additional allowed regions; ~a, ~b, ~l, ~p: generously allowed regions; From A to a to ~a, the rationality of dihedral angle decreases in turn (**B**) Z-score of the predicted structure compared with the Z-scores of the experimental structures from different sources (X-ray and NMR); in the database, it is considered as a more reasonable structure if its Z-score falls within the range of the experimental structures. (**C**) Energy of all residues calculated by PROSA and the black line represents the average energy of residues, which is calculated by taking a 40-residue window. A positive value means that the position of the corresponding residue is abnormal. The green line represents the energy of all residues calculated by PROSA and the black line represents the average energy of residues, which is calculated by taking a 40-residue window. (**D**) 3D/1D score of the predicted structure calculated by VERIFY_3D and the blue line represents the average score, which is the standard to judge the quality of the structure; it is considered as a high-quality model when more than 80% of the residues have 3D/1D scores higher than 0.2.

We used Alphafold2 to predict the Q875I9 sequence with the signal peptide already removed (221 residues in total) (Figure 1A). The predicted lDDTs of all residues except several loop regions were above 95 (Figure 1B), so the confidence of the structure was high. In the model evaluation, PROCHECK was first used (Figure 2A), and 89.4% of the rotation corners were in the most favored regions. This result is close to the high quality judging criteria (90%), indicating that the angles formed between residues are reasonable. Next, we used VERIFY_3D to evaluate the prediction (Figure 2B), and the results showed that the average 3D/1D scores of all residues exceeded 0.2 (the minimum value was 0.28) and most of them exceeded 0.5, indicating that the compatibility of the sequences with the structure was fully conformed to the requirement of high quality. Finally, we used PROSA to evaluate, from the energy perspective, the Z-score falls within a reasonable range of NMR and X-ray crossover (Figure 2C), and the mean energy values of all residues were all negative, meeting the criteria of high quality (Figure 2D). Therefore, the predicted structure passed all of the evaluations, proving that it is reasonable.

### 2.2. Structural Analysis and Catalytic Residues

**Figure A2:**
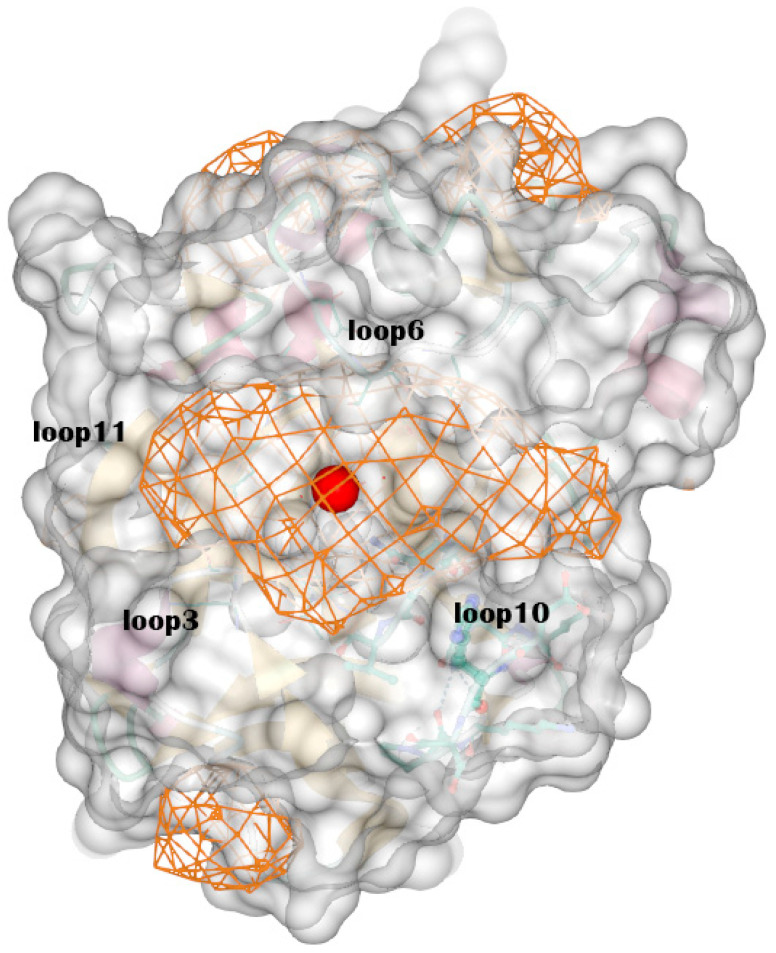
The optimal binding site the DeepSite found. It is surrounded by loop 3, loop 6, loop 10 and loop 11. The chitosanase is shown by transparent surface and the possible binding sites are shown by grid. The red ball represents the center of binding site.

The predicted structure can be divided into a total of seven α-helices, six β-sheets, and 14 loops (Table A1), according to the criteria of Pymol. Using DeepSite to find binding sites in the structure, the coordinates of the center of three sites with the highest scores point to the same site, which consist of some residues in loops 3, 6, 7, 10, and 11, while the opening of the region consists of residues in loops 3, 6, 10, and 11 (Figure A2). The protonation states of the residues were determined using H++ at pH 6.5 (Table A2).

ASP143 and GLU152 are two catalytic residues where the pKa have been experimentally validated, and Cheng predicted their roles in catalysis based on the experience that ASP143 may have a smaller pKa than GLU152 and tends to be deprotonated, so it should act as a generalized base in catalysis (gaining a proton), while GLU152 tends to be protonated and acts as a generalized acid [12]. However, the calculation of H++ showed that the pKa of ASP143 increased from 3.86 in neutral to 8.53, while GLU152 only increased from 4.25 to 6.85, so the roles of both in catalysis are exactly the opposite of what was predicted. The pK_decomposition file output by H++ quantifies the extent to which the neighboring residues of each residue contribute to its pKa change. By analyzing the interaction terms between the titratable groups (Table A3), we found a special relationship existing among ASP61, ASP63, TYR103 and ASP143, that is, the top three residues that affected the pKa of each residue were just the other three of these four, and most values were above 2.0. It can be inferred that these four polar or charged residues may have formed a more stable interaction network, thus making it more difficult for the protons to be dissociated, leading to a significant increase in pKa. None of the values contributing most to GLU152 exceeded 1.6, so its pKa did not increase much. This is an inference made based on the structure, which needs to be verified later by relevant experiments.

**Figure 3:**
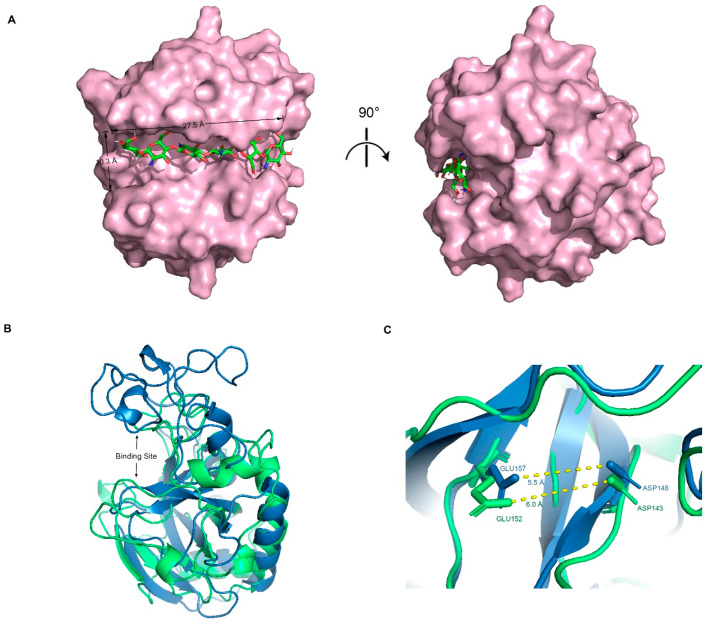
(**A**) Complex conformation after molecular docking, where the chitosanase is shown by the surface and the DP6-chitosan is shown by the stick, and the view of the right side was rotated 90° to show the side view. (**B**) Q875I9 was structurally aligned with V-Csn by Pymol (green represents Q875I9 and blue represents V-Csn). (**C**) Magnified display of the relative position of two catalytic residues in both two chitosanases, respectively, and the yellow dashed lines represent the linear distance between the respective catalytic residues.

The optimal docking result between DP6-chitosan and the binding site was obtained (Figure 3A). We found that the length of the binding site (about 27.5 Å) was roughly the same as that of DP6-chitosan, and the narrowest width (about 10.3 Å) was enough to allow chitosan to enter and interact with the surrounding residues. Moreover, the catalytic residues ASP143 and GLU152 were located almost at the center of the binding site, and the distance between their side chains was only about 6.0 Å (Figure 3C). If the glycosidic bond only moves to the middle of them, the enzymatic reaction can easily occur. Because members from the same family tend to be structurally similar, we aligned its structure with the experimental structure of V-Csn from the soil virus AMG [19]. Although their sequences are quite different, they still share a high similarity in overall structure, with the RMSD of only 1.33 Å. More importantly, their binding sites are not only similar in structure and size (Figure 3B), but also have almost the same positions and relative distances of catalytic residues (Figure 3C). This result demonstrates the accuracy of the predicted structures from both the geometric and evolutionary perspectives.

### 2.3. Stability of Simulation System

**Figure 4:**
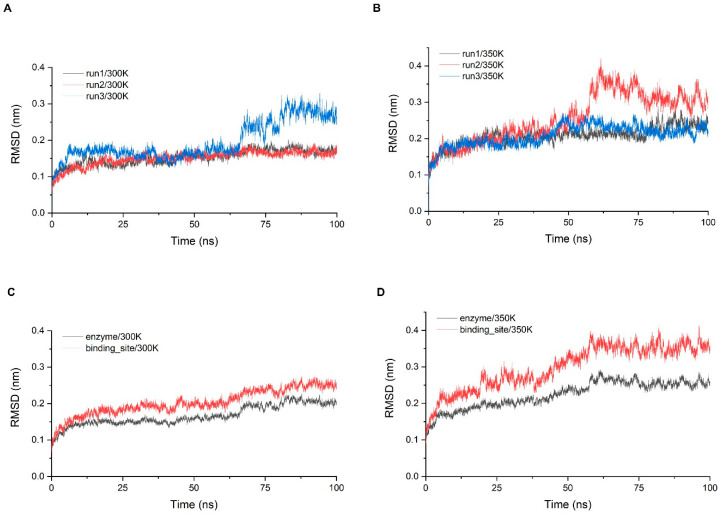
(**A**,**B**) The RMSD of the three simulations at 300 K and 350 K; (**C**,**D**) show the comparisons of the RMSD average values between the whole structure and binding site at each temperature.

Stability analysis was performed for three simulations of 100 ns at 300 K and 350 K, respectively. The system stability was first monitored by calculating the RMSD of the Cα atom with time (Figure 4A,B). While all trajectories gradually entered the equilibrium state after 10 ns, some equilibrium states were disrupted later and re-entered other equilibrium states, which took place at both temperatures, but more frequently at 350 K. The RMSD of the trajectories at 300 K were concentrated around 0.15 nm for 90% of the time and the fluctuations were small. In addition, the last 30 ns of the third simulation showed a large fluctuation of more than 0.1 nm, indicating that significant conformation changes still occurred with a small probability. The RMSD of the trajectories at 350 K were greater than 0.2 nm for more than half of the time, indicating that the fluctuation of overall structure was a little greater. To examine the stability of the critical sites, we screened all residues within 5 Å of chitosan based on the molecular docking results and defined that these residues comprise the binding site of the enzyme (Figure A7). We examined the RMSD of Cα in the binding site range and calculated the average of three simulations and then compared it with the average of the RMSD of the overall structure (Figure 4C,D). The RMSD of the binding site was higher than the overall one at both temperatures, indicating that the binding site has a tendency to fluctuate more than the overall structure. At 300 K, the index was only about 0.05 nm higher than the overall which was within the normal range, while at 350 K, there was a significant increase of about 0.1 nm, so the stability of the binding site was much lower than that of the overall structure at high temperature.

**Figure 5:**
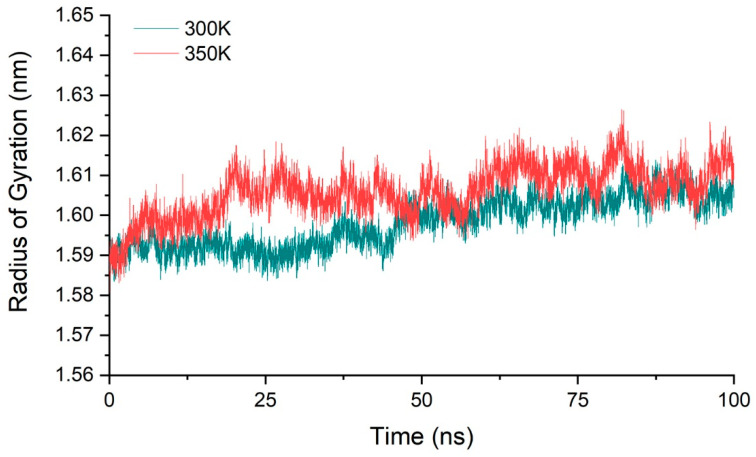
The average radius of gyration of three simulations at 300 K and 350 K.

We calculated the molecular radius of gyration of chitosanase for three simulations at 300 K and 350 K, respectively, and their average value to ensure that all samples were fully considered (Figure 5). The result showed that the radius varied within 1.58–1.62 nm at both temperatures, with a range of only 0.04 nm. The mean value of the gyration radius at 350 K only increased by about 0.01 nm compared with 300 K, less than 1% compared with the radius scale of 1.6 nm. This showed that the overall structure of the enzyme is relatively stable during the heating process, and there was no tendency of protein denaturation such as looseness or swelling, so the conformational changes may only be located in a small part of the region. This also provides a good structural basis for the next step of enzyme modification.

**Figure 6:**
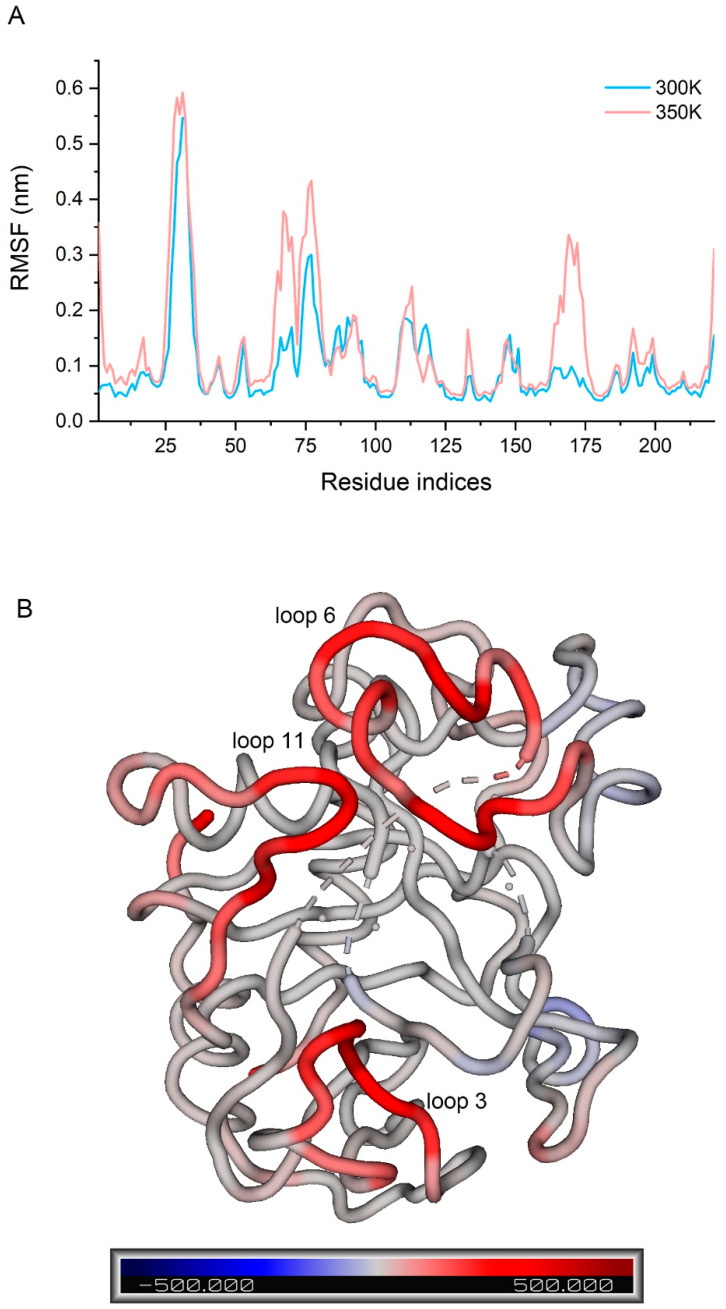
(**A**) RMSF of the concatenated trajectories at 300 K and 350 K; (**B**) difference in B-factor between 300 K and 350 K in the structure by spectrum.

To fully consider all the conformations that had been sampled, we concatenated the trajectories of the three simulations at each temperature, then calculated the RMSF of Cα atoms in the two concatenated trajectories and compared them (Figure 6A). It has been shown that the difference in RMSF between systems with different temperature can be used to indicate the flexibility of a protein or a region to temperature [29]. We found that loop 3 had the highest flexibility at 300 K (over 0.5 nm) followed by loop 6 (over 0.3 nm), while loops 7 and 10 also fluctuated to some extent (below 0.2 nm). Flexibility at 350 K was only significantly higher than 300 K in a few regions rather than across the board. Loop 3 still had the largest fluctuation and the flexibility of several residues in loop 3 showed a significant increase. Some regions in loop 6 and 11 showed a large increase, with some residues increasing by more than 0.2 nm. Loop 11, in particular, changed from the low fluctuation region to the third highest fluctuation region, so it is likely that the residues causing the decrease in enzyme activity stability are in these regions. The B-factor was calculated for each residue at both temperatures, and a higher B-factor value represents higher flexibility in the corresponding region [30]. The difference values were displayed in the structure by spectrum (Figure 6B), allowing for good visualization of the change in flexibility. We screened the residues with a great change in flexibility (not less than 0.1 nm in difference values and not including both ends) and defined them as the highly flexible region (Figure A7).

### 2.4. Dynamic Cross Correlation Analysis

**Figure 7:**
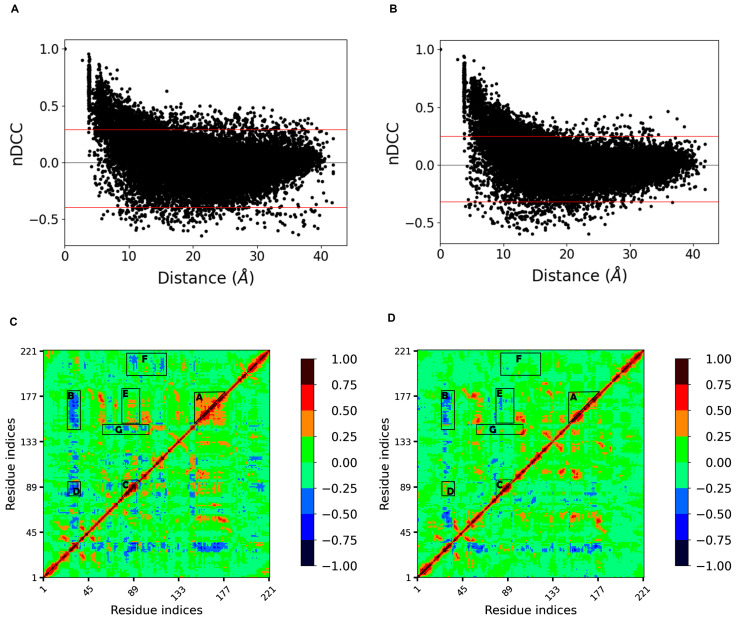
(**A**,**B**) show the distributions of the correlation indices with increasing distance between residues at 300 K and 350 K, respectively; the distance between two red lines shows the thickness of the boat-shaped distribution, and the distance narrowed at 350 K, indicating a decreasing trend of both positive and negative correlations (closer to 0); (**C**,**D**) show the cross correlation matrix diagrams at 300 K and 350 K, respectively.

Correlationplus [31] was used to perform dynamic cross correlation analysis on the concatenated trajectories at 300 K and 350 K, respectively. The distribution of the correlation indices with increasing distance between residues was first represented by scatter plots (Figure 7A,B). We found that at both temperatures, the distributions were similar and were both boat-shaped: the quantity of negative correlations gradually increased and eventually exceeded the positive correlations as the distance increased; both gradually decreased to zero. However, the thickness of the boat at 350 K was significantly smaller than that at 300 K (i.e., the absolute values of many correlation indices decreased at equal distance), indicating that the correlation between residues decreases and the disorder of motion increases at high temperature.

The cross correlation matrix diagram (Figure 7C,D) showed that the regions with strong extended positive correlations on the diagonal had negative correlations with loop 3 at 300 K. Loops 10 and 11, corresponding to region A with higher internal positive correlations, showed higher negative correlations with loop 3 (region B), while loop 6 and helix 3, corresponding to region C, also had significant negative correlations with loop 3 (region D). These four regions correspond to the four loops that make up the binding site (i.e., the whole binding site has a stable motion correlation at room temperature). After heating up to 350 K, the internal positive correlation between regions A and B was significantly reduced, so the negative correlation between region A as a whole and loop 3 disintegrated into several parts, and the negative correlation between region B and loop 3, together with the adjacent sheet 1 and helix 2, almost disappeared. This suggests that high temperature weakened the interactions within regions A and B and decreased the corresponding correlations. Loops 6 and 11, represented by region E, showed a certain positive correlation at 300 K, which was due to their spatial proximity and the interaction between them. However, this correlation disappeared at 350 K, indicating that the motion correlation between them no longer exists. Dynamic cross correlation networks have been shown to be critical for conformation change and ligand binding [32,33]. These changes in the correlations appearing around the binding site may indicate a dramatic change in the conformation. In addition, the regions represented by regions F and G with correlation at 300 K almost disappeared at 350 K, probably also due to the increased disorder of residue-motion. This analysis confirmed the highly flexible region previously determined by RMSF, and narrowed down the range of residues that affect thermal stability.

### 2.5. Major Conformation Analysis

**Figure 8:**
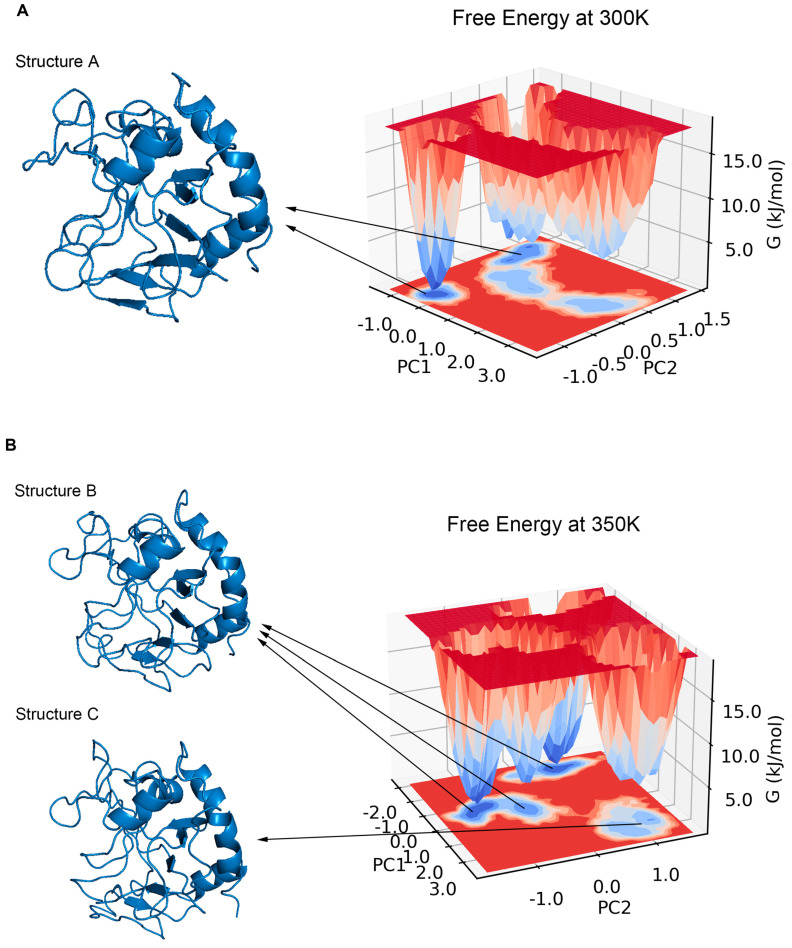
(**A**) shows the free energy landscape with two principal components at 300 K, all energy basins corresponded to the first structural cluster (structure A). (**B**) shows the free energy landscape with two principal components at 350 K, the three lowest basins corresponded to the first structural cluster (structure B), and the other basin corresponded to the second structural cluster (structure C).

**Figure A3:**
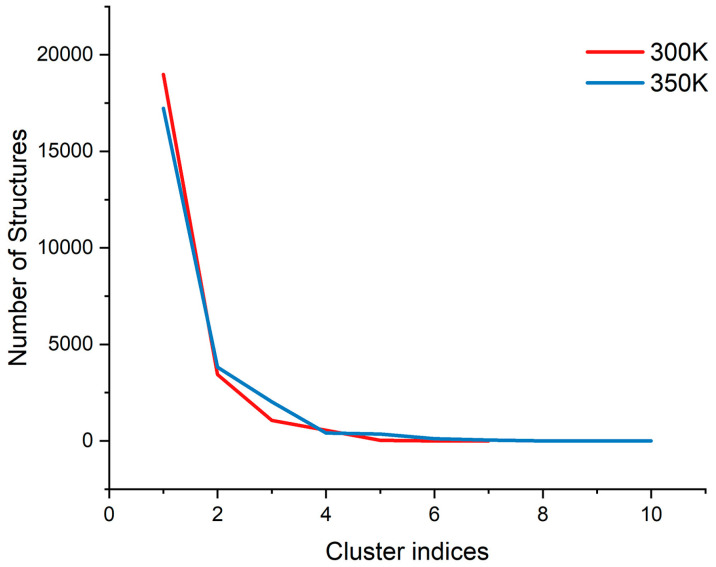
The trend of the number of structures with the cluster indices at each temperature.

PCA has been shown to be able to identify and classify a large number of conformations in trajectories to help find the dominant structure [34]. We calculated the PCA for the trajectories at 300 K and 350 K, respectively. The results showed that the first two principal components could explain more than 55% of the molecular motion at 300 K and nearly 44% at 350 K. Plotting the free energy surface can help us search the lowest energy conformation [35]. In the free energy landscape (Figure 8), there was only one free energy basin at 300 K with a relative free energy of −13.419 KJ/mol, and the energy of the second minimal point was already 2 KJ/mol higher; the lowest valley at 350 K had a relative free energy of −18.492 KJ/mol, while there was another basin with a free energy only less than 0.05 KJ/mol higher. If one energy basin corresponds to multiple conformations, cluster analysis can be used to find the most dominant conformation among them [36,37]. We yielded seven and 10 structural clusters at 300 K and 350 K, respectively. The first cluster accounted for 78.8% of all samples in the trajectory at 300 K and 71.8% at 350 K, both being the dominant conformations in their respective environments (Figure A3). We also found the representative conformation of the main structural clusters corresponding to the minimum energy points (Figure 8): at 300 K, the deepest free energy basin in the landscape corresponded to the first structural cluster (structure A); at 350 K, the two deepest free energy basins were actually similar in terms of the overall structure, and together corresponded to the first structural cluster (structure B), while another basin with a higher free energy at a farther distance corresponded to the second structural cluster (structure C), accounting for 16%.

**Figure 9:**
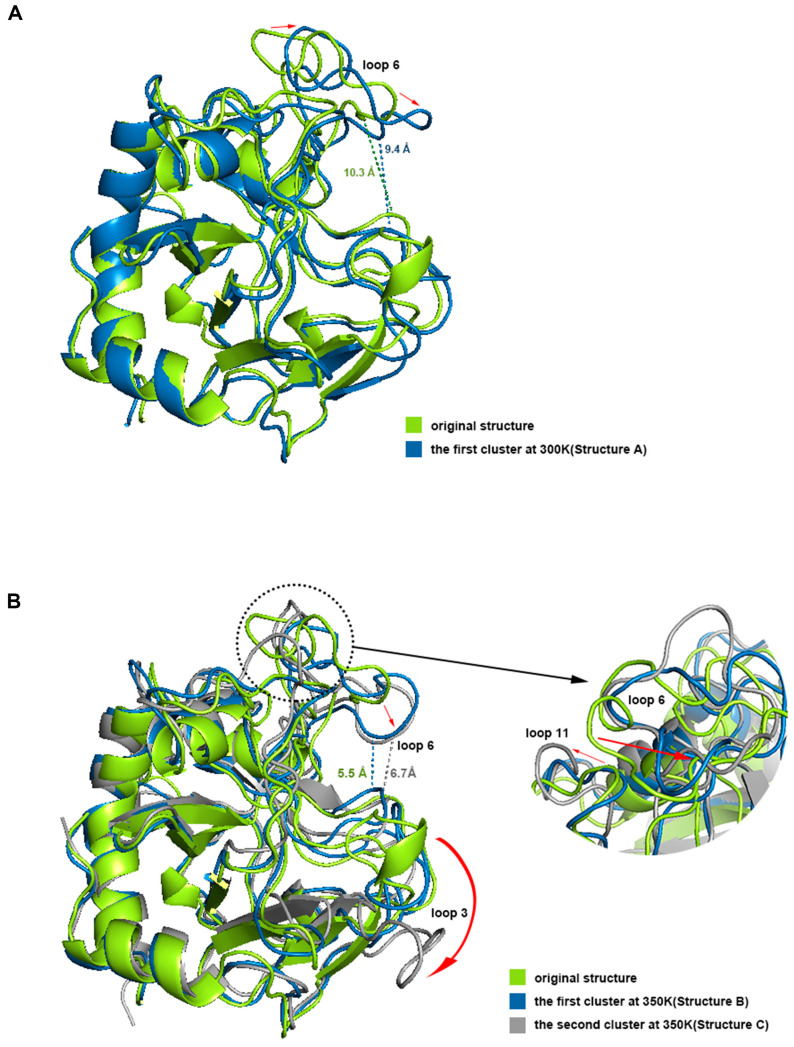
(**A**,**B**) shows the aligning of the dominant conformations with the initial structure at 300 K and 350 K respectively. The dotted lines represent the distance at the narrowest positions of the conformational openings and the red arrows represent the motion trends of residues.

We aligned structures A, B, and C with the initial structure (Figure 9). The results showed that the opening of the binding site in loop 6 sunk a little at 300 K, but the narrowest width of the opening only dropped from 10.3 Å to 9.2 Å, which did not affect the binding with chitosan at all. Loop 3 and loop 11 also had some deviation. However, the difference between structure A and the initial structure was not significant from the overall view, especially the binding site, which still maintained the same structure and size as the original one, which is consistent with the results of the previous RMSF analysis. Compared with the initial state, the most significant change in structure B is that the opening of the site within loop 6 sunk significantly, and the narrowest width was reduced to 5.5 Å. Because the opening was transformed into a flat lying state, the effective vertical distance directly opposite the catalytic site was only about 3.8 Å. This structure showed a closed conformation, which makes it impossible to accommodate the chitosan molecule. Structure C at 350 K did not change much in terms of the size of the opening compared to structure B, but loop 3 shifted significantly outward, completely leaving the position of the binding site in the initial state, which may adversely affect the binding of chitosan. Meanwhile loop 11 showed a large outward shift, which resulted in the distance between the closest residue pair, with loop 6 being pulled away from 4.2 Å to 10.1 Å. This may be the reason for the significant decrease in the positive correlation between loop 6 and loop 11 in the above dynamic cross analysis. The above analysis shows that the conformations of loops 3, 6, and 11 all changed a lot at high temperature, and these changes, especially the change of opening from open to closed, will hinder the binding with the ligand.

### 2.6. Residues Interaction Analysis

**Figure 10:**
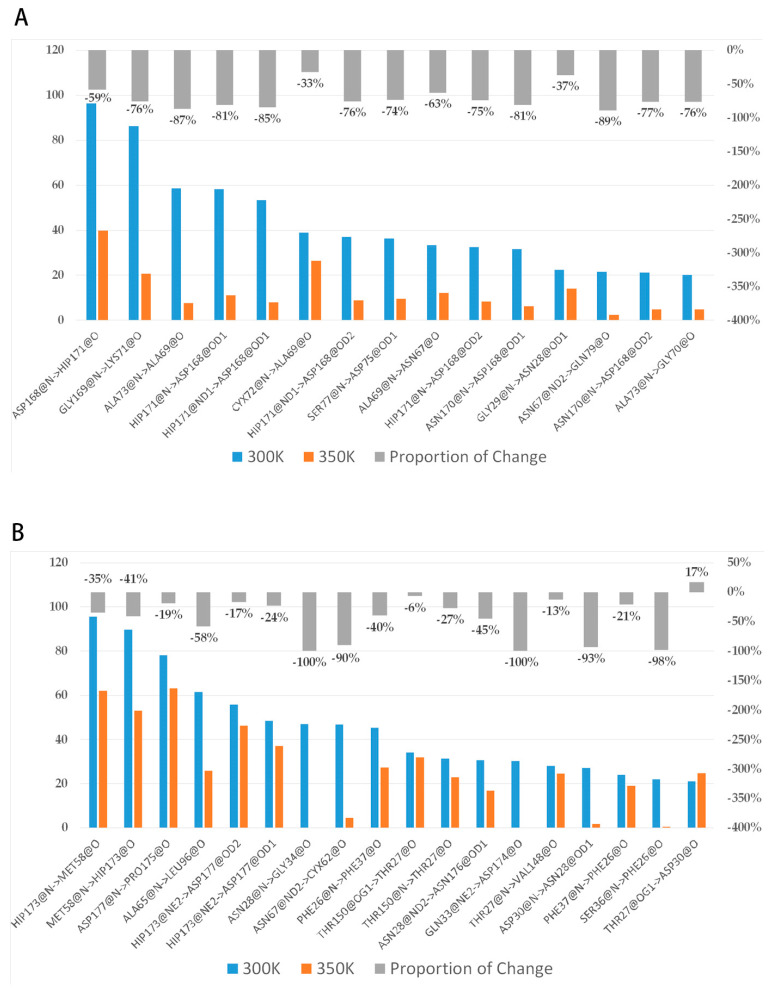
(**A**,**B**) show the change in probability of intra-group and inter-group H-bonds, respectively. The blue-orange bar charts represent the proportion of important hydrogen bonds that occur, and the gray bar charts represent the percentages of changes in the proportions relative to themselves after heating up.

**Figure A4:**
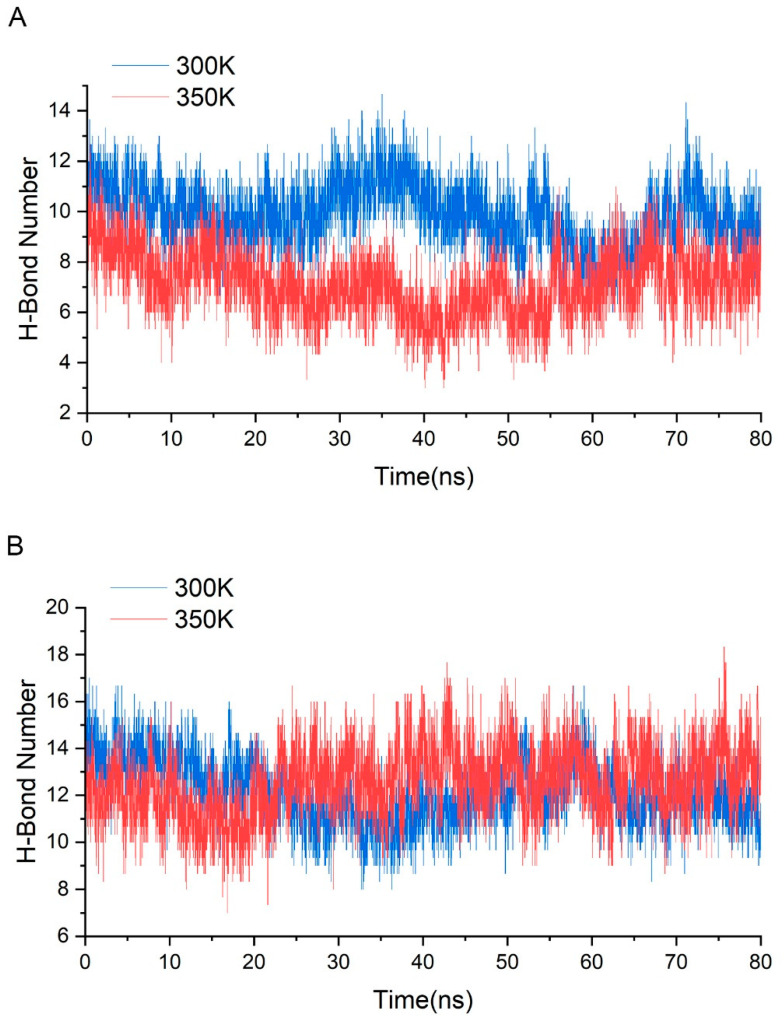
(**A**) shows the number of intra-group H-bonds and (**B**) shows the number of inter-group H-bonds in simulations at 300 K and 350 K.

To investigate the reasons for conformational change, we analyzed the interactions among residues. Many studies have confirmed that hydrogen bonds make a very important contribution to the stability of protein [38,39], so we first grouped the highly flexible region separately, then took the last 80 ns equilibrium trajectories at two temperatures and calculated the average of intra-group (between group members) and inter-group (out-group residues) hydrogen bonds for three simulations. The trend in the number of both hydrogen bonds over time was counted (Figure A4). It was found that the number of intra-group hydrogen bonds decreased significantly after heating up, with the average value dropping from 10 to near 7; the number of inter-group hydrogen bonds was close to 12–13, and the high temperature did not have a significant effect on it. This difference may be related to the hydrogen bond breaking and forming. Hydrogen bonds with a probability of occurrence of not less than 20% at 300 K were screened and the change in probability after heating up was calculated (Figure 10A). The results showed that there were 15 important hydrogen bonds in the group, and the probability of appearing after heating up showed a precipitous drop, with all hydrogen bonds dropping by more than 30%, and 73% of them dropping by more than 70%. This indicated that there is a certain correlation between the level of thermal stability and the increase in hydrogen bonds. The intensification of molecular motion brought by high temperature severely damaged the intra-group hydrogen bonds, so their ability to maintain the region stability was significantly reduced correspondingly. The decrease in the inter-group hydrogen bonds was not as drastic as that of the intra-group, only about 56% of the hydrogen bonds dropped by more than 30% (Figure 10B). In addition, the intra-group hydrogen bonds were all from the same secondary structure, in other words, the residue pairs generating hydrogen bonds were relatively close in sequence, while the proportions of some inter-group ones across multiple secondary structures were highly variable such as HIP173-MET58 (from loop 11 and loop 6), GLN33-ASP174, and ASN28-ASN176 (from loop 3 and loop 11), ALA65-LEU96 (from loop 6 and loop 7), etc. These hydrogen bonds make contributions to maintain the relative positions between secondary structures, and the breaking implies that a high temperature leads to a significant change in the relative positions.

The above analysis was reversed to analyze the level of hydrogen bond forming. The probabilities of hydrogen bonds with a probability of not less than 20% after heating up were counted at room temperature (Table 1); the brand new ones and those with a significant increase in probability were included in the statistics. Aside from three new inter-group hydrogen bonds, there were six new intra-group hydrogen bonds with 50% across the secondary structures. This may be caused by changes in the relative positions among secondary structures, and these new ones likewise contribute to the stability of the new conformation. In contrast, the relative position changes of the highly flexible region both break old hydrogen bonds and form new ones, which is an important reason why the number of inter-group hydrogen bonds does not change significantly after heating up.

**Figure 11:**
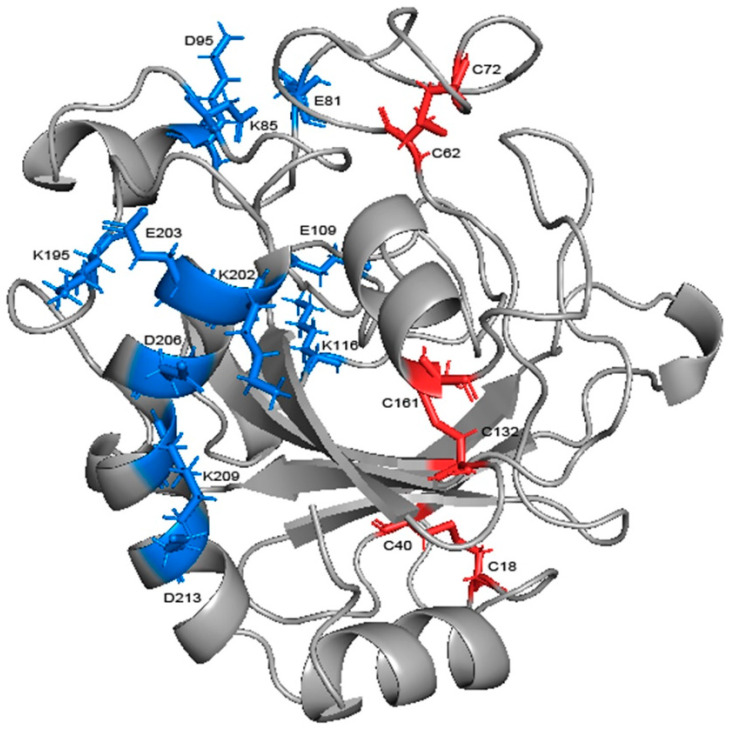
The salt bridges and disulfide bonds in the structure of chitosanase. The blue sticks represent salt bridges and the red ones represent disulfide bonds.

Salt bridges in the protein were calculated at each temperature, those with a probability of occurrence of not less than 20% were similarly screened and the change in probability was compared (Table 2). There were six salt bridges with high stability in total, among which GLU109-LYS116, LYS209-ASP213, and LYS202-ASP206, were from loop 7 and helix 7, respectively, and accordingly, GLU81-LYS85 connects loop 6 with helix 3, LYS85-ASP95 connects helix 3 and loop 7, and LYS195-GLU203 connects loop 13 and helix 7 (Figure 11). Four salt bridges exceeded 20% at 300 K; three of them had a decrease in probability but none of them exceeded 20%, while the other one had a rise of more than 25% after heating up. Moreover, the high temperature caused a significant increase in the probability of the other two salt bridges. This indicates that high temperature does not cause obvious damage to the salt bridge. Five of them were located in the more stable α-helix. Except for GLU81-LYS85 (16% decrease only), all of them were far from the binding site and thus protected from the highly flexible region. Helix 7, consisting of 18 residues, is the largest helical region and an important backbone in the structure. LYS209-ASP213, located on it, had the highest percentage (74%) of the entire simulation, and together with LYS202-ASP206, further reinforced the skeleton. The three salt bridges linking the α-helix region to the surrounding loops are loosely structured, so also contribute to reinforcing the overall structure as well as GLU109-LYS116, which can reduce the flexibility of loop 7. Salt bridges have been identified as one of the main factors contributing to thermal stability in glycoside hydrolases [40,41,42]. Therefore, the stability at high temperature means that salt bridges play a role in stabilizing the overall structure.

**Figure 12:**
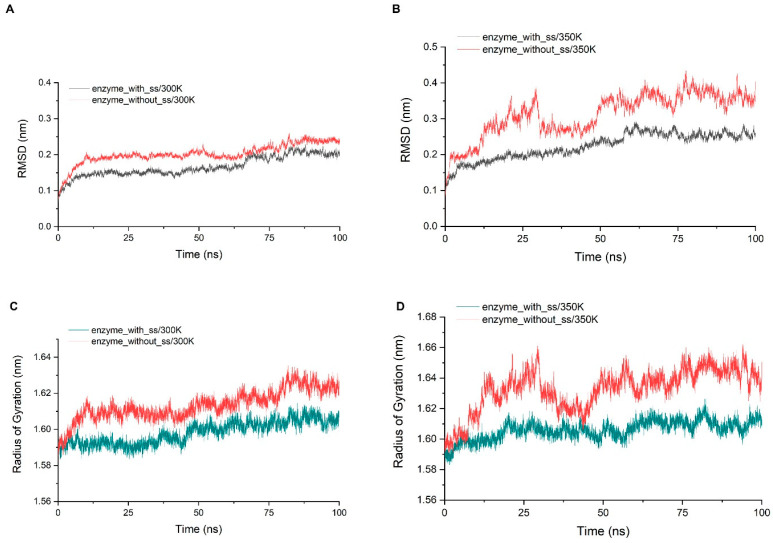
(**A**,**B**) show the comparison of the RMSD average values of three simulations at 300 K and 350 K between the chitosanase with and without disulfide bonds; (**C**,**D**) show the comparison of the average of the radius of gyration of three simulations at 300 K and 350 K between the chitosanase with and without disulfide bonds.

**Figure A5:**
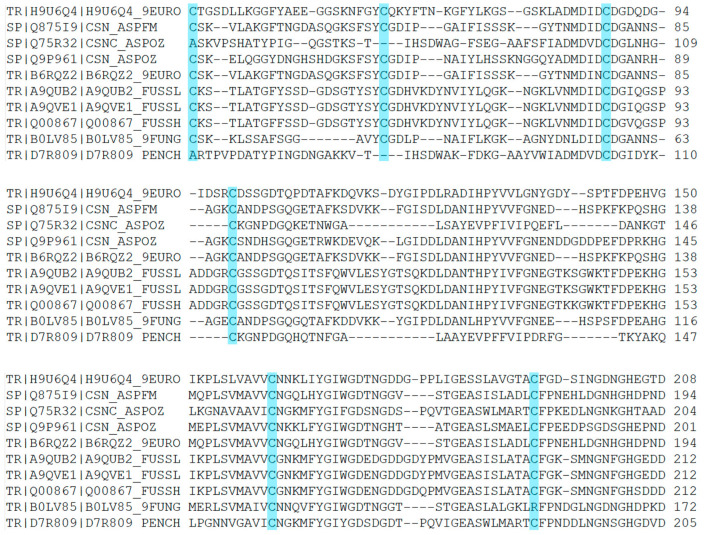
Sequence alignment and cysteine conservation in the family GH75.

Through the alignment of sequences from GH75 (not including virus source), we found that all six cysteines in sequence were highly conserved, indicating that their existence may be important for the structure and function of the enzyme (Figure A5). In addition, the distances within each cysteine pair (18–40, 62–72, 132–161) were very close, and the SG–SG distances of all three groups were no more than 2.5 Å, which is in accordance with Amber’s disulfide bond criteria. In order to investigate the effect of disulfide bonds on the stability, the chitosanase model without disulfide bonds was simulated three times at both 300 K and 350 K, and the mean of the RMSD and radius of gyration were calculated and compared with the one with disulfide bonds (Figure 12). The RMSD increased at both temperatures after the loss of disulfide bonds and much more at 350 K (above 0.1 nm), which indicates that the stability decreased; the radius of gyration also increased at both temperatures and more at 350 K (above 0.03 nm), indicating that the volume became larger and the structure had an obvious tendency to loosen. Therefore, the disulfide bonds play an important role in maintaining the stability of the structure. Studies have shown that the GH45 and GH75 enzymes bear a strong structural similarity [19], and disulfide bonds play a vital role in the thermostability of cellulase from GH45 [43]. Considering all of these analyses, it can be reasonably inferred that three disulfide bonds should exist (Figure 11). CYX62–CYX72 are located inside loop 6, which have an important role in stabilizing the binding site. CYX18–CYX40 and CYX161–CYX132 firmly connect the core region containing six β-sheet layers to the outer, so the whole structure always has a pull toward the center, ensuring that the structure will not easily tend to loosen. This is consistent with the above analysis of the radius of gyration. In summary, the disruption of the hydrogen bonds inside and outside the highly flexible region, and the formation of new hydrogen bonds after heating up should be the main interaction factor for the conformational changes in the binding site, while the good stability of salt bonds and the existence of disulfide bonds explain why the overall structure is so stable at high temperature.

### 2.7. Binding Free Energy Analysis

**Figure 13:**
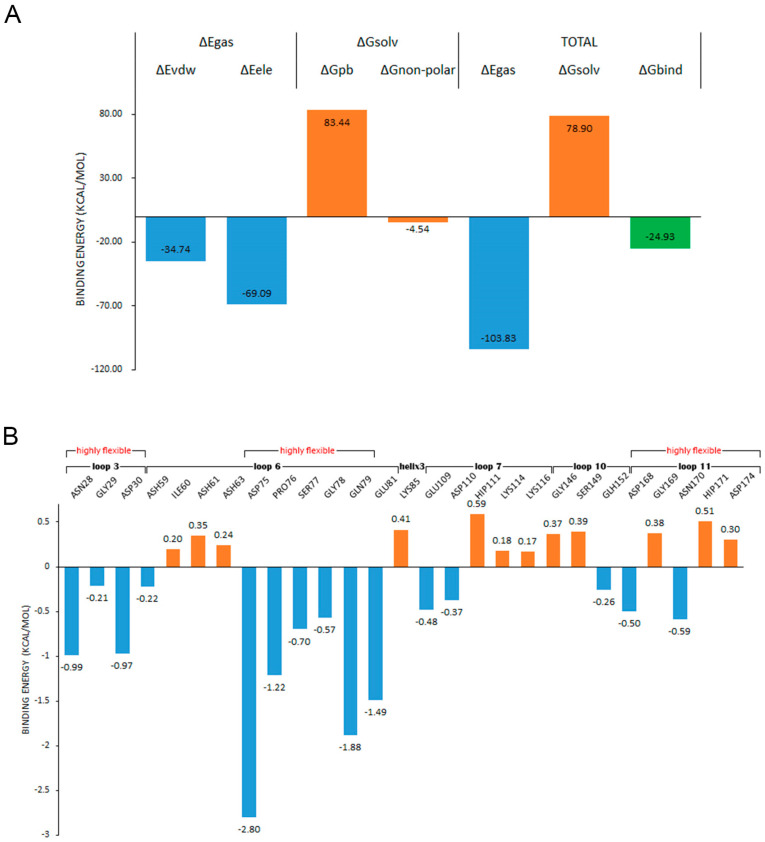
(**A**) shows the compositions of the binding free energy; (**B**) shows the decomposition of the binding energy on the residues and the second structures and highly flexible regions that these residues belong to.

**Figure A6:**
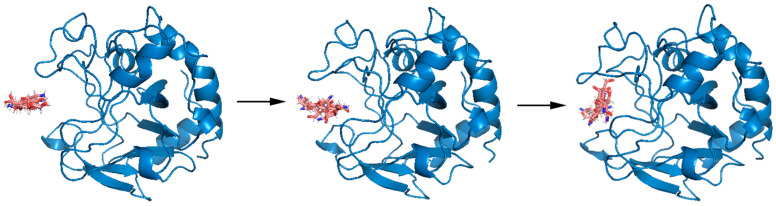
A typical binding process of chitosanase and DP6-chitosan at 300 K.

**Figure A7:**
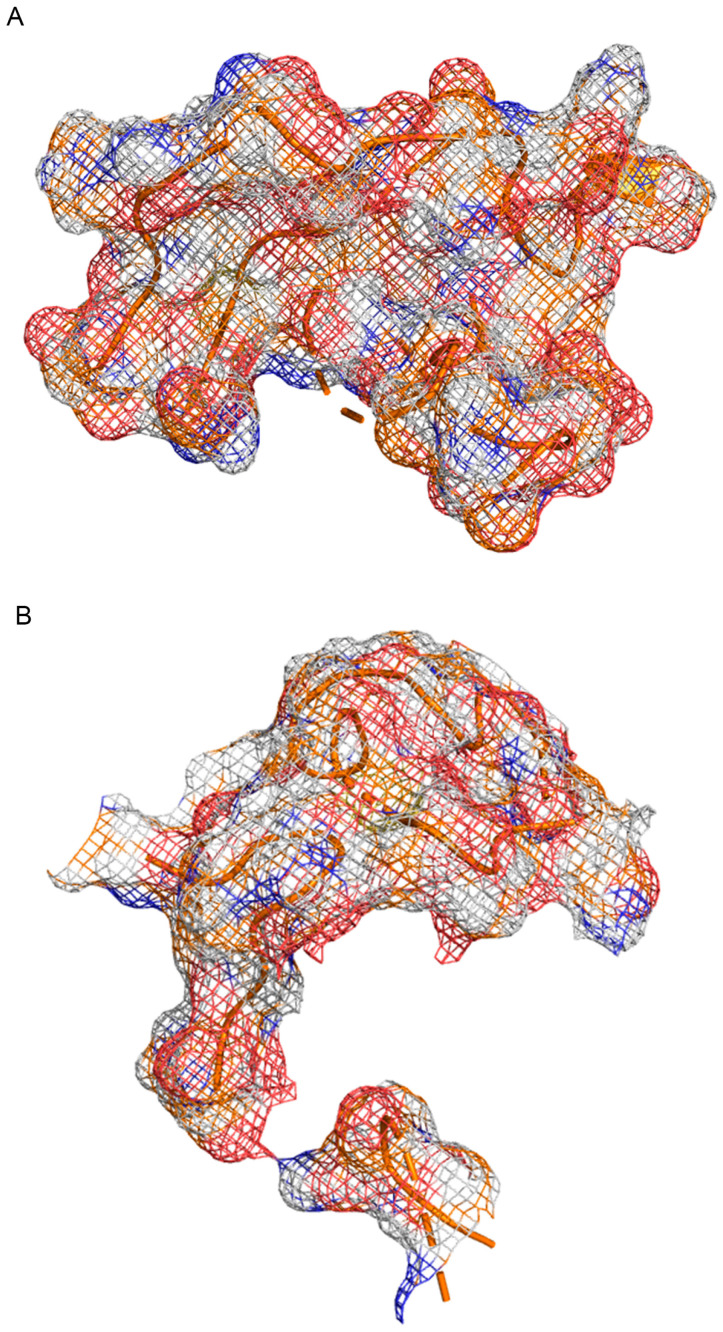
(**A**,**B**) show the grouping of binding site and highly flexible regions respectively.

MMPBSA has been widely applied as an efficient and reliable free energy simulation method to model molecular recognition such as for protein–ligand binding interactions [44]. In order to determine the influence of the fluctuation difference of the highly flexible region on the stability of the enzyme, we investigated the contribution of each residue to the binding. The ligand was placed about 8 Å away from the center of the binding site, and the binding simulation was performed three times at 300 K for 50 ns. The ligand entered the binding site in every simulation, and kept a stable binding conformation with chitosanase during the simulation time (Figure A6). The average binding free energy was calculated by taking the stable last 30 ns trajectory to ensure that all samples were fully considered. In the compositions of binding free energy (Figure 13A), the total binding energy was −24.93 kcal/mol, which indicated that chitosanase had a good binding ability with DP6-chitosan. The main contribution to the binding force came from the molecular mechanical energy in which the contribution of electrostatic interaction was more than twice that of the VDW force. The obstacles encountered in the binding all came from the polar part of solvation energy (PB), which was as high as 83.44 kcal/mol, indicating that the interaction between molecules was enough to overcome the resistance of the solvation effect. After decomposing the binding energy into residues (Figure 13B), we found that four residues with the lowest binding energy all came from loop 6, and the energy of two residues in loop 3 was also close to −1 kcal/mol. Although there were some residues in loops 7, 10, 11 and the second half of loop 6 that promoted the binding, the number of residues that hindered the binding and the corresponding energy were larger, so the overall effect was negative. Most of the contributing residues were located at the opening position of the binding site, which pull DP6-chitosan from outside into the site. ASH59 and GLH152, located inside the site, also promoted the stable binding. Comparing the contributing region with the highly flexible region revealed that there was a high consistency between them, and all of the contributing residues in loop 3 and loop 6 belonged to the highly flexible region. Table A4 shows the flexibility (RMSF difference) of the residues whose contributions were less than −0.5 kcal/mol. Except for GLU81, the flexibility of other residues was obviously higher than the average, and 70% of them reached or exceeded three times that of the average, indicating that the flexibility of the contributing residues was significantly higher than that of the whole. However, it is these highly flexible residues whose binding energy accounted for 78.7% of all. This must lead to the contributing residues not being stable enough in the correct position, which will greatly reduce the binding capacity of the site. Therefore, the high flexibility of contributing residues as well as the closure of site should be the main reasons for the significant decrease in the thermal stability of chitosanase at high temperature.

## 3. Materials and Methods

### 3.1. Structural Prediction and Model Evaluation

The prediction accuracy of the AlphaFold 2 model from Deep-mind is close to the experimental error, and this study used the online version of AlphaFold 2 on the Colab platform [45]. In addition to the predicted structure, the model also provides a confidence score for each residue (predicted lDDT), taking values ranging from 0 to 100. Above 90 means that the confidence of the residue is very high, between 70 and 90 means that it is confident, between 50 and 70 means that it is low confidence, and below 50 means that the confidence is very low. The structure with the highest score will be selected in the prediction results.

Three widely used model evaluation tools—PROCHECK, VERIFY_3D, and PROSA—were used to evaluate the prediction results. PROCHECK [46,47] evaluates the stereo chemical parameters of the 3D structure. The allowed and disallowed regions were defined by the psi (ψ) and phi (φ) of a peptide chain and the results are shown by Ramachandran plots. VERIFY_3D [48,49] determines the compatibility between the amino acid sequence and its 3D structure by assigning the structure class and comparing it with good structures. PROSA [50,51] regards the total energy of protein as a function of sequence and conformation, and converts it into the Z-score, which is then compared with the Z-scores of the experimental structures in the database. In addition, the energy is also taken as a function of the residue indices to measure whether each residue is reasonable or not.

### 3.2. Protonation, Binding Site Search, and Molecular Docking

The optimum pH and the pKa value of fully deacetylated chitosan are both 6.5, so all protonation operations were carried out at pH 6.5. The protonation of chitosan was calculated to be 50% based on the relationship between them (Equation (A1) in Appendix A), so three amino groups of DP6-chitosan were protonated for simulation. Protonation of the chitosanase was performed using the online server H++ [52,53,54]. After we found the most likely binding site with DeepSite [55], the docking of chitosanase to chitosan was carried out using Autodock Vina version 1.2.0 [56,57].

### 3.3. Molecular Modeling and Molecular Dynamics Simulation

The enzyme-only and enzyme-DP6 simulation systems were modeled using Ambertools22 suite [58,59]. The chitosanase can cleave linkages of GlcN–GlcN and GlcNAc–GlcN [12] and mainly convert DP6-chitosan into DP3-chitosan without significant activity on DP5-chitosan or shorter oligosaccharides [16]. Moreover, the complex conformation of V-Csn and DP6-chitosan with 100% of deacetylation has been successfully obtained [19]. Therefore, the degree of polymerization of chitosan was set to 6 and the deacetylation degree was set to 100%. The AMBER-ff19SB force field [60] was used for chitosanase and GAFF2 [61] was used for DP6-chitosan, then the ions were added to maintain the electrical neutrality. The OPC water model [62] was used to construct a cubic water box to solvate the systems, and the minimum distance of the solute from the edge of the box was 10 Å. Experiments have shown that the ff19SB force field combined with the OPC model can have a better prediction ability [60]. All of the simulations were modeled with periodic boundary conditions.

All simulations were carried out by the GPU version of Gromacs2022.1 [63]. In the pre-equilibrium stage, the steepest descent method was used to minimize the energy. Under the constant pressure of 1 bar, the temperature was gradually heated from 0 K to the target temperature in 1000 ps and given sufficient time for relaxation. The equilibrium was ensured by monitoring the potential energy and temperature convergence of the system at each stage. Then, the production phase simulations were carried out for 50 to 100 ns. The SETTLE algorithm [64] was used to constrain the structure of water, while the LINCS algorithm [65] was used to constrain the bonds connected to H atoms. The particle mesh Ewald (PME) algorithm [66] was used to estimate the long-range electrostatic interaction, the V-rescale algorithm was used to couple the temperature of the solute and solvent to achieve more accurate control, and the Berendsen algorithm [67] and Parrinello-Rahman algorithm [68] were used to couple the pressure of the system at NPT and the production phase, respectively. The enzyme-only system was used to carry out three production phase simulations at each temperature to ensure sufficient sampling, and the enzyme-DP6 system was used to carry out three production phase simulations at 300 K to ensure that the calculation of the binding free energy was more accurate.

### 3.4. Analytical Methods

#### 3.4.1. Conformational Fluctuation Analyses

The root mean square deviation (RMSD) (Equation (A2) in Appendix A) reflects the fluctuation of the structure of the target molecule over time. The root mean square fluctuation (RMSF) (Equation (A3) in Appendix A) reflects the average variation of residues, and can be translated into a B-factor (Equation (A4) in Appendix A) reflected in the protein structure. Both the RMSD and RMSF were calculated for the Cα atom of the enzyme in this study. The radius of gyration (Equation (A5) in Appendix A), as a rough measure of structural compactness, reflects the change in volume and shape of the molecule over time, from which it can be determined whether the protein has a tendency to loosen and denature.

#### 3.4.2. Dynamic Cross Correlation

Dynamic cross correlation calculates the correlation of motion trends between atoms in a structure and is very effective for identifying long-range interactions as well as key residues, etc. We found the correlated motion trends between residues by calculating the mutual correlations between Cα atoms (Equation (A6) in Appendix A).

#### 3.4.3. Principal Component Analysis, Free Energy Landscape and Cluster Analysis

Principal component analysis (PCA) is a widely used method for the dimensionality reduction in data. The covariance matrix is first calculated from the coordinate trajectories, and the eigenvectors and eigenvalues obtained after the diagonalization reflect the direction and the motion in that direction, respectively. Then, the free energy landscape along the direction is calculated to obtain the relative free energy surface for the entire trajectory (Equation (A7) in Appendix A). Next, the gromos algorithm [69] was used to cluster different conformations in a trajectory according to a specific algorithm, thus dividing a large number of conformations into different classes and finding the most dominant ones.

#### 3.4.4. Interaction Analysis

The hydrogen bonds were calculated to analyze the interactions among residues. Whether the hydrogen bonds existed were determined using the following geometrical criterion: the distance between the donor heavy atom D and the acceptor heavy atom A is not greater than 3.5 Å and the angle formed by H-D-A is not greater than 30°. The salt bonds of the enzyme were calculated using VMD’s “saltbr” plugin [70], and the probability of each salt bond occurring across the whole trajectory was counted using a self-written script. The geometric criterion for the salt bonds is that the distance between the center of mass of the O atom in the acidic side chain and the center of mass of the N atom in the basic side chain is not greater than 4 Å.

#### 3.4.5. Binding Free Energy Analysis

Compared with other algorithms for binding free energy, MMPBSA can better balance the computational cost and accuracy, and also decompose the binding energy to specific residues. We used gmx_MMPBSA [71] to calculate and assign the binding free energy.

## 4. Conclusions

In this study, we predicted the chitosanase from *Aspergillus fumigatus Y2K* by Alphafold2 and found that the structure of the binding site and the position of catalytic residues were highly similar to those of V-Csn from GH75, which proved the reliability of this prediction. In contrast to the predictions from the previous literature, the structure-based protonation predictions predicted that ASP143 may act as a generalized acid in catalysis.

After heating up to 350 K, although the overall structure remained relatively stable as at 300 K, the flexibility of the binding site increased significantly and the motion correlation between certain secondary structures decreased, which indicates that the high temperature makes the conformation of the critical region unstable. PCA and cluster analysis revealed that the dominant conformation of the binding site changed from an open to closed state, which spatially hindered the binding to the ligand. The main reason for the conformational change is that hydrogen bonds belonging to the highly flexible region are destroyed at 350 K. The intense molecular motion caused by high temperature means that they no longer play a role in maintaining the stability. The location of the salt bonds, where they have linking effects, and their good stability at high temperature allow them to make an important contribution to the overall stability along with the three disulfide bonds, which can connect the core region to the outer.

From the point of view of the binding free energy, the contributing residues were mainly distributed in the opening of the binding site, and were highly consistent with the highly flexible region. Therefore, the intensification in the fluctuation will inevitably lead to a decline in binding capacity, which will eventually reduce the stability of enzyme activity. This conclusion provided the potential sites for modification. A close and stable interaction network can help enzymes resist the interference of the environment on their structures [72] and can be considered to increase the residue interactions around the highly flexible region by mutation to improve the stability of the binding site. Research methods based on the predicted structures provide the possibility of research on a large number of proteins without experimental structures, allowing computational methods to show their magic in this field.

## Figures and Tables

**Table 1:** The hydrogen bonds with a probability of not less than 20% after heating up in both the intra-group and inter-group as well as the difference and percentage of change between before and after heating up.

Type	Hydrogen Bond (Donor->Acceptor)	300 K (%)	350 K (%)	Difference (%)	Percentage (%)
Intra-Group	GLY64@N->GLN79@O	3.18	31.77	28.59	899
	HIP171@N->ASP168@O	0.06	22.54	22.48	37,400
	CYX72@N->SER68@O	4.5	25.69	21.19	471
Inter-Group	GLN79@NE2->GLU81@O	0.64	34.1	33.46	5228
	ALA65@N->GLY80@O	0	30.79	30.79	brand new
	LYS35@N->ALA31@O	0.03	30.39	30.36	101,200
	ILE60@N->ASN170@O	0	24.33	24.33	brand new
	PHE26@N->GLY34@O	0	22.82	22.82	brand new
	ASN57@ND2->HIP173@O	6.01	26.49	20.48	341

**Table 2:** The salt bridge with a probability of not less than 20% before and after heating up as well as the difference and percentage of change between before and after heating up.

Salt Bridge	300 K (%)	350 K (%)	Difference (%)	Percentage (%)
GLU81-LYS85	36.60	30.64	−5.95	−16.27
LYS85-ASP95	19.73	30.30	10.57	53.60
GLU109-LYS116	34.29	43.00	8.70	25.38
LYS195-GLU203	50.74	45.75	−4.99	−9.83
LYS202-ASP206	4.01	20.23	16.22	404.25
LYS209-ASP213	87.28	73.61	−13.66	−15.65

## Data Availability

Data is contained within the article.

## Appendix A

(A1)
$$P_{\mathrm{protontatd}}=\frac{10^{pK_{a}-pH}}{1+10^{pK_{a}-pH}}$$

(A2)
$$\mathrm{RMSD}=\left(t_{1},t_{2}\right)={\left[\frac{1}{M}\sum_{i=1}^{N}m_{i}{∥r_{i}\left(t_{1}\right)-r_{i}\left(t_{2}\right)∥}^{2}\right]}^{1/2}$$

(A3)
$$RMSF=\sqrt{\frac{1}{\mathrm{nsteps}}\sum_{i=1}^{nsteps}{∥r_{i}(t)-\langle r_{i}\rangle ∥}^{2}}$$

(A4)
$$B\text{-}\mathrm{factor}=RMSF^{2}\left(\frac{8}{3}\right)\pi^{2}$$

(A5)
$$R_{g}={\left(\frac{\sum_{i}{∥r_{i}∥}^{2}m_{i}}{\sum_{i}m_{i}}\right)}^{1/2}$$

(A6)
$$C_{ij}=\frac{\langle r_{i}\cdot r_{j}\rangle -\langle r_{i}\rangle \langle r_{j}\rangle }{\sqrt{\left[\left(\langle r_{i}^{2}\rangle -{\langle r_{i}\rangle }^{2}\right)\left(\langle r_{j}^{2}\rangle -{\langle r_{j}\rangle }^{2}\right)\right]}}$$

(A7)
$$G_{a}=-kT\ln\left(\frac{P\left(q_{a}\right)}{P_{\max}(q)}\right)$$

## Appendix C

**Table A1:** shows the specific residue divisions of secondary structures.

Classification	Residue Composition
helix1-7	ASN5-HID14, ALA31-GLN33, LYS85-PHE91, PRO117-HIE120, ILE155-CYS161, LYS186-ALA188, ALA201-GLY218
sheet1-6	LEU22-PHE26, PHE37-CYS40, ALA46-SER50, LEU125-CYS132,GLN135-TRP141, LEU179-PHE183
loop1-14	TYR1-PRO4, LYS15-VAL21, THR27-ASP30, GLY34-SER36, GLY41-GLY45, SER51-PHE84, GLY92-LYS116, GLY121-PRO124, ASN133-GLY134, GLY142-SER154, PHE162-VAL178, THR184-SER185, VAL189-ASN200, LEU219-ALA221

**Table A2:** shows the change of protonation states at pH 6.5.

Original	Protonated	Residue Number
ASP	ASH	59, 61, 63, 143
GLU	GLH	152
HIS	HID	14
HIS	HIE	101, 120
HIS	HIP	111, 137, 166, 171, 173

**Table A3:** shows the first three neighbors with the greatest influence on the pKa change of each residue and the quantized value of influence.

Residue	ASP61			ASP63			TYR103			ASP143			GLU152		
Neighbours	103	63	61	61	103	143	61	63	143	61	103	63	61	59	103
Value	4.09	2.36	2.18	2.36	2.02	1.73	4.09	2.02	1.92	2.18	1.92	1.73	1.58	1.19	0.98

**Table A4:** shows the residues with binding energy contribution less than −0.5 kcal/mol, and the corresponding RMSF differences, as well as the average of differences of all residues.

Binding Energy (kcal/mol)	Residue	RMSF Difference(nm)	Average of Differences(nm)
−2.80	ASP75	0.07	0.04
−1.88	GLN79	0.12	
−1.49	GLU81	0.03	
−1.22	PRO76	0.13	
−0.99	ASN28	0.2	
−0.97	ASP30	0.07	
−0.70	SER77	0.13	
−0.59	ASN170	0.22	
−0.57	GLY78	0.15	
−0.50	ASP168	0.21	
